# Association of clozapine with structural and resting-state functional abnormalities of the hippocampus in chronic schizophrenia

**DOI:** 10.3389/fpsyt.2024.1464066

**Published:** 2024-10-04

**Authors:** Sung Woo Joo, Sang Kyoung Kim, Won Hee Lee, Se Hyun Kim, Jungsun Lee

**Affiliations:** ^1^ Department of Psychiatry, Asan Medical Center, University of Ulsan College of Medicine, Seoul, Republic of Korea; ^2^ Department of Software Convergence, Kyung Hee University, Yongin, Republic of Korea; ^3^ Department of Psychiatry, Seoul National University Hospital, Seoul National University College of Medicine, Seoul, Republic of Korea

**Keywords:** schizophrenia, clozapine, hippocampus, cognitive function, functional connectivity

## Abstract

**Introduction:**

Abnormalities in the hippocampus have been extensively reported in schizophrenia research. However, inconsistent findings exist, and how structural and functional abnormalities of the hippocampus are associated with clinical symptoms in schizophrenia, especially concerning clozapine treatment, remains uncertain.

**Methods:**

We recruited 52 patients with schizophrenia, each with an illness duration of at least 5 years, and categorized them based on clozapine treatment. T1-weighted images and resting-state functional magnetic resonance imaging scans were obtained and analyzed to perform group comparisons of the structural and functional changes in the hippocampus. Volumes of the hippocampal subregions, as well as resting-state functional connectivity maps from these areas were compared between the groups. Associations with clinical symptoms, including the severity of psychiatric symptoms and cognitive functions, were investigated.

**Results:**

The clozapine group (n=23) exhibited smaller volumes in several hippocampal subregions, including the CA1, CA4, granule cell and molecular layers of the dentate gyrus, compared to the non-clozapine group (n=29). Seven clusters with significant group differences in functional connectivity with these hippocampal subregions were identified, with six of these clusters showing increased functional connectivity in the clozapine group. The reduced volumes of the hippocampal subregions were moderately associated with the severity of negative symptoms, general intelligence, and executive function.

**Discussion:**

Patients with schizophrenia undergoing clozapine treatment exhibited smaller volumes in the hippocampal subregions, which were moderately associated with negative symptoms and cognitive functions, compared to those without clozapine treatment.

## 1 Introduction

Despite extensive neuroimaging studies on structural abnormalities in schizophrenia, the clinical utility of these findings is still limited. Previous large-sample studies (1, 2) indicate structural abnormalities in schizophrenia, including global reductions in cortical thickness, particularly in frontal and temporal regions, and volume reductions in the hippocampus, amygdala, thalamus, and accumbens. However, robust group-level abnormalities are scarcely represented at the individual level (3). Wolfers et al. showed that only a few regions had an overlap of structural abnormalities in > 2% of patients with schizophrenia, indicating heterogeneity among patients (3). This heterogeneity may be linked to clinical heterogeneity in terms of psychiatric symptoms, responses to antipsychotic treatment, and long-term illness courses (4).

Patients with a long duration of schizophrenia have more similar clinical characteristics than those with first-episode psychosis. Indefinite antipsychotic treatment is recommended for patients with multiple episodes of schizophrenia (5) due to a higher risk of relapses, especially upon discontinuation of medication. These patients tend to experience a decline in cognitive functions (6, 7) as well as social and occupational functioning (8). Investigating brain abnormalities in patients with chronic schizophrenia could reveal disease-specific abnormalities that might be obscure in clinically heterogeneous populations such as those with first-episode or recent-onset psychosis.

Treatment resistance, indicated by insufficient responses to more than two different antipsychotic treatments, is characterized by poor improvement in symptoms and functioning. Although various definitions of treatment resistance have been used (9), evidence suggests clozapine as the most effective antipsychotic for treatment-resistant schizophrenia (10). Clozapine’s unique receptor profile may contribute to its superior effectiveness, leading to discussions about separating patients with treatment-resistant schizophrenia from those with treatment-responsive schizophrenia from a neurobiological perspective. However, mixed results persist (11, 12). A systematic review of neuroimaging studies highlighted differences such as decreases in gray matter and perfusion in frontotemporal regions and increases in perfusion in white matter and basal ganglia in treatment-resistant versus treatment-responsive schizophrenia (13). The authors suggested the need for further studies to elucidate the neurobiological characteristics of treatment-resistant schizophrenia due to limited replication of findings.

According to Lieberman et al. (52), hippocampal dysfunction is evident from the prodromal to later stages of schizophrenia. In the prodromal stage, attenuated psychotic symptoms are associated with elevated neuronal activity due to glutamate neurotransmission dysregulation in the Cornu Ammonis (CA)1 region of the hippocampus. As the illness progresses, dysfunction extends within and beyond the hippocampus, resulting in atrophic changes. Decreased hippocampal volume has been reported in individuals at high risk for psychosis (14, 15) and early psychosis (16, 17). In chronic schizophrenia, hippocampal volume loss is more pronounced (2); however, whether the volume reduction is progressive remains unclear (18, 19). A longitudinal study indicated progressive hippocampal volume loss in treatment-resistant schizophrenia after clozapine treatment initiation (20). Regarding functional dysconnectivity, Kraguljac et al. showed that resting-state functional connectivity of the hippocampus with other regions, including the anterior cingulate cortex, caudate nucleus, auditory cortex, and calcarine sulcus, predicts response to antipsychotic medications in unmedicated patients (21). Reduced functional connectivity between the hippocampus and the striatum has been observed in patients with schizophrenia and their family members (22). McHugo et al. reported anterior hippocampal hyperactivity in early psychosis, indicating ineffective recruitment during a 1-back scene-processing task (23). These findings support the structural and functional abnormalities of the hippocampus in schizophrenia, though few studies have investigated the associations with treatment resistance and/or clozapine treatment.

Here, we investigated the structural and resting-state functional abnormalities of the hippocampus in patients with long-term schizophrenia. Participants were categorized by clozapine use at the magnetic resonance imaging (MRI) scan, and group comparisons of structural and functional abnormalities were conducted. We hypothesized that 1) the clozapine group would exhibit lower hippocampal volumes compared to the non-clozapine group, 2) the reduced volumes would vary across hippocampal subregions, and 3) differences in resting-state functional connectivity between the hippocampal subregions and other brain regions would be observed. We also examined associations with clinical variables, including the severity of psychiatric symptoms, cognitive functions, and general functioning.

## 2 Methods

### 2.1 Study population

We recruited participants from the Asan Medical Center, a university-affiliated hospital, from June 2021 to December 2023. The inclusion criteria were: 1) a schizophrenia diagnosis according to the Diagnostic and Statistical Manual of Mental Disorders V (DSM-V); 2) illness duration of > 5 years; and 3) right-handedness. Participants were excluded based on the following criteria: 1) a history of intellectual disability; 2) a history of substance abuse or dependency in the last 6 months; 3) a history of head trauma leading to > 3 min of unconsciousness; 4) neurological disorders; and 5) unstable diseases that may interfere with brain functioning. Interviews and evaluations of the participants were conducted 1 week before the MRI scan, and clinical information including previous and current illness history, family history, and antipsychotic treatment history was obtained. The severity of psychiatric symptoms was assessed using the Positive and Negative Syndrome Scale (PANSS) (24), and cognitive functions were measured by means of a shorter version of the Wechsler Adult Intelligence Scale-Fourth edition (WAIS-IV), Rey-Kim Memory Test, and Kims Frontal-Executive Neuropsychological Test. All participants provided written informed consent before enrollment. A total of 69 patients with schizophrenia were enrolled, with 14 withdrawing consent, resulting in 55 patients who completed the study protocol. This study received approval from the IRB of the AMC (IRB No. 2021-1128, 2022-1193) and was conducted according to the Declaration of Helsinki.

The final study population consisted of 52 patients with schizophrenia, excluding three due to poor image quality (n=2) and major neurological malformation (n=1). We grouped the study population based on whether they were on clozapine treatment at the time of the MRI scan. During the interview, we confirmed that there was no history of clozapine treatment in the non-clozapine group except for one patient who had taken clozapine a few years prior and had not taken the medication since. Given several limitations of the current study, including its cross-sectional design, the relatively long duration of illness, and possible recall error for symptom severity, we categorized the study population based on current clozapine treatment rather than treatment resistance. **Table 1** presents the demographic and clinical characteristics of the final study population. The duration of illness between the two groups was not statistically different. The clozapine group exhibited higher PANSS total (t=-2.523, p=0.015), positive subscale (t=-3.480, p=0.002), and general subscale (t=-2.076, p=0.043) scores, as well as lower General Assessment of Functioning (GAF) (t=3.065, p=0.004) and Executive Function Quotient (EFQ) (t=2.332, p=0.024) scores compared to the non-clozapine group.

**Table 1:** Demographic and clinical characteristics of the study population.

Variable	Non-clozapine	Clozapine	t or χ^2^	p value
Number of participants	29	23		
Age, mean (SD), years	41.0 (11.5)	36.2 (13.1)	1.397	0.169
Sex, male, n (%)	15.0 (51.7)	11.0 (47.8)	<0.001	1.000
Duration of illness, mean (SD), years	17.2 (9.8)	14.7 (8.6)	0.957	0.343
Clozapine treatment				
Treatment duration, mean (SD), years	NA	8.2 (7.2)		
Daily dose, mean (SD), mg/day		232.6 (112.9)		
PANSS				
Total, mean (SD)	59.7 (19.9)	73.0 (17.2)	-2.523	0.015
Positive subscale, mean (SD)	12.6 (4.4)	16.9 (4.5)	-3.480	0.002
Negative subscale, mean (SD)	17.2 (7.0)	20.3 (5.4)	-1.741	0.088
General subscale, mean (SD)	29.9 (10.6)	35.8 (9.5)	-2.076	0.043
GAF, mean (SD)	67.1 (11.1)	58.0 (9.7)	3.065	0.004
FSIQ, mean (SD)	92.7 (18.3)	87.2 (13.3)	1.187	0.241
MQ, mean (SD)	76.8 (16.1)	68.1 (15.0)	1.933	0.059
EFQ, mean (SD)	83.1 (19.0)	70.9 (17.7)	2.332	0.024

SD, standard deviation, PANSS, positive and negative syndrome scale, GAF, global assessment of functioning, FSIQ, full scale intelligence quotient, MQ, memory quotient, EFQ, executive function quotient.

### 2.2 Image acquisition, preprocessing, and analysis

A 32-channel dStream head coil at 3T Philips Ingenia CX was used for the MRI scans. The parameters of T1-weighted images and resting-state functional MRI (fMRI) are provided in **Supplementary Table 1**. During the resting-state fMRI scan, participants were instructed to maintain a steady head position, focus their gaze on a central crosshair, and avoid thinking of anything in particular.

Visual inspection of the T1-weighted images and resting-state fMRIs excluded three participants due to poor image quality (n=2) and major neurological malformation (n=1).

T1-weighted images were analyzed using the FreeSurfer (version 7.4) automated pipeline (http://surfer.nmr.mgh.harvard.edu/fswiki/recon-all). The pipeline included skull stripping, volumetric labeling, intensity normalization, tessellation of the boundary between the gray and white matter, automated topology correction, and surface deformation. We selected seven subcortical regions in each hemisphere: accumbens, amygdala, caudate, hippocampus, thalamus, pallidum, and putamen. The hippocampus module (25) in FreeSurfer was used to obtain volumes of hippocampal subregions, including parasubiculum, presubiculum, subiculum, CA1, CA3, CA4, GC-ML-DG (Granule Cell and Molecular Layer of the Dentate Gyrus), molecular layer, Hippocampal Amygdala Transition Area (HATA), fimbria, hippocampal tail, and hippocampal fissure.

The preprocessing and denoising of resting-state fMRI were performed with SPM12 (Wellcome Trust Centre for Neuroimaging, London, UK) and the CONN toolbox v22a (http://www.nitrc.org/projects/conn). We followed the default preprocessing pipeline, which included functional realignment and unwarp, slice-timing correction, outlier detection, direct segmentation and normalization, and functional smoothing. We excluded eight participants whose number of outlier scans, identified during preprocessing, exceeded the third quartile + 1.5-times the interquartile range. Consequently, 44 patients were included in the functional connectivity analyses. Denoising of the preprocessed resting-state fMRIs was performed according to the default denoising pipeline in the CONN toolbox. Potential confounding effects were regressed out separately for each voxel and participant using a general linear model (GLM). Potential noise effects were included as first-level covariates in the GLM, including signals from white matter and ventricles (5 components), 12 motion parameters, and outlier scans. Functional smoothing was performed using spatial convolution with a Gaussian kernel of 8 mm full width at half maximum. A temporal band-pass filter of 0.008–0.09 Hz was applied. Detailed preprocessing and denoising procedures are described elsewhere (26). We performed a seed-to-voxel analyses using the hippocampus and its subregions in both hemispheres as seed regions, defined according to the Harvard–Oxford atlas (FMRIB Software Library, Oxford, UK) and the Julich Brain Atlas v3.1 (27), respectively. Mean time series from each seed region were extracted and used to create functional connectivity maps. Functional connectivity values in the maps were converted into z-values for group-level statistical inferences.

### 2.3 Statistical analysis

Student’s t- or chi-square tests were used to compare continuous or categorical variables in the demographic and clinical characteristics of the participants.

A multiple linear regression model was used to compare volumes of the subcortical regions and hippocampal subregions between the non-clozapine and clozapine groups. Age, sex, and estimated intracranial volume were included as covariates in the model. Correction for multiple comparisons was performed based on a false discovery rate (FDR) q of 0.05, accounting for the number of subcortical regions or hippocampal subregions.

Resting-state functional connectivity with the seed regions was compared between the non-clozapine and clozapine groups using the GLM with covariates of age and sex. Voxel-wise uncorrected p of 0.005 and cluster size-based FDR p-value of 0.05 were used to adjust for multiple comparisons.

Associations with clinical symptoms were assessed using the PANSS total, positive subscale, negative subscale, general subscale, GAF, full-scale intelligence quotient (FSIQ), memory quotient, and EFQ scores. We included the volumes of hippocampal subregions and mean functional connectivity of clusters with the seed regions that showed significant group differences. Spearman’s rho was used for testing associations with clinical variables, with correction for multiple comparisons based on the FDR q of 0.05, accounting for the number of clinical variables (n=8). Further, associations between illness duration and the clozapine dose were examined with multiple corrections for the number of hippocampal subregions or clusters.

Statistical significance was determined using an alpha value of 0.05. All statistical analyses were performed using R software (version 4.0.2; R Development Core Team, Vienna, Austria), with the exception of the resting-state functional connectivity analyses, which were conducted using the CONN toolbox v22a.

## 3 Results

### 3.1 Volumes of hippocampal subregions in the non-clozapine and clozapine groups

**Supplementary Table 2** shows the results of group comparisons of the volumes of subcortical regions between the non-clozapine and clozapine groups. The clozapine group had lower volumes of the left (t=-3.009, FDR p=0.029) and right (t=-3.063, FDR p=0.029) hippocampus than the non-clozapine group. **Table 2** presents the volumes of hippocampal subregions in the non-clozapine and clozapine groups. The volumes of CA1 (left, t=-3.677, FDR p=0.015; right, t=-3.170, FDR p=0.016), CA4 (left, t=-2.764, FDR p=0.024; right, t=-2.829, FDR p=0.029), GC-ML-DG (left, t=-2.643, FDR p=0.033; right, t=-2.877, FDR p=0.024), and the molecular layer (left, t=-3.245, FDR p=0.016; right, t=-3.174, FDR p=0.016) in both hemispheres were decreased in the clozapine group compared with the non-clozapine group.

**Table 2:** Volumes of hippocampal subregions in the non-clozapine and clozapine groups.

Region	Non-clozapine	Clozapine	t	Uncorrected p	FDR p	η_p_^2^
	Volume^a^					
Left CA1	644.2 (85.7)	566.0 (56.0)	-3.677	0.001	0.015	0.248
Left CA3	203.9 (27.2)	186.6 (25.6)	-1.970	0.055	0.092	0.092
Left CA4	527.7 (67.4)	482.4 (44.7)	-2.764	0.008	0.024	0.179
Left fimbria	89.3 (26.4)	85.0 (19.5)	-1.320	0.193	0.232	0.053
Left GC-ML-DG	283.0 (37.8)	259.0 (24.3)	-2.643	0.011	0.033	0.159
Left HATA	52.7 (10.6)	47.5 (8.3)	-1.987	0.053	0.092	0.110
Left hippocampal fissure	139.2 (34.8)	130.6 (19.3)	-0.452	0.654	0.682	0.006
Left hippocampal tail	540.3 (83.5)	497.8 (81.9)	-1.845	0.071	0.104	0.076
Left molecular layer	557.6 (72.6)	501.5 (42.3)	-3.245	0.002	0.016	0.210
Left parasubiculum	59.3 (12.3)	53.0 (12.9)	-1.945	0.058	0.092	0.100
Left presubiculum	307.2 (51.6)	289.2 (39.4)	-1.380	0.174	0.220	0.064
Left subiculum	446.1 (64.7)	407.4 (40.9)	-2.433	0.019	0.050	0.138
Right CA1	688.0 (89.2)	619.2 (60.7)	-3.170	0.003	0.016	0.203
Right CA3	215.3 (29.5)	204.0 (21.0)	-1.382	0.174	0.220	0.056
Right CA4	542.8 (67.1)	498.6 (40.9)	-2.829	0.007	0.029	0.168
Right fimbria	89.0 (26.9)	86.6 (18.1)	-0.911	0.367	0.400	0.027
Right GC-ML-DG	292.1 (36.9)	267.8 (22.0)	-2.877	0.006	0.024	0.182
Right HATA	54.0 (10.9)	48.6 (10.4)	-1.981	0.053	0.092	0.109
Right hippocampal fissure	145.2 (32.4)	140.3 (20.8)	-0.128	0.899	0.899	0.001
Right hippocampal tail	536.9 (74.1)	517.9 (60.2)	-1.200	0.236	0.270	0.041
Right molecular layer	578.8 (72.2)	526.3 (44.9)	-3.174	0.003	0.016	0.206
Right parasubiculum	53.7 (12.8)	47.9 (11.8)	-1.828	0.074	0.104	0.081
Right presubiculum	287.8 (58.1)	259.3 (39.9)	-2.039	0.047	0.092	0.105
Right subiculum	446.6 (64.0)	408.8 (42.3)	-2.267	0.028	0.067	0.119

a Volumes of hippocampal subregions are presented as mean (SD).

CA, cornu ammonis; GC-ML-DG, granule cell and molecular layer of the dentate gyrus; HATA, hippocampal amygdala transition area ;FDR, false discovery rate.

### 3.2 Resting-state functional connectivity with the hippocampal subregions in the non-clozapine and clozapine groups

Resting-state functional connectivity with the hippocampus seed regions was compared between the non-clozapine and clozapine groups. The cluster located in the right supramarginal gyrus showed increased functional connectivity with the left hippocampus seed region in the clozapine group compared to the non-clozapine group (**Supplementary Figure 1**). The clozapine group showed increased functional connectivity of the right hippocampus seed region to the cluster located in the anterior cingulate gyrus compared to the non-clozapine group (**Supplementary Figure 2**).

**Figure 1:**
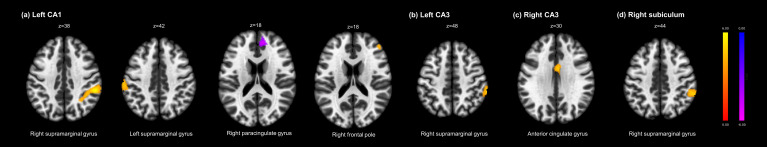
Group differences in resting-state functional connectivity with the hippocampal subregions between the non-clozapine and clozapine groups. **(A)** Left CA1, **(B)** Left CA3, **(C)** Right CA3, **(D)** Right subiculum. The yellow to red and blue to purple bars indicate t-values for the increase and decrease in resting-state functional connectivity in the clozapine group, respectively. Clusters were overlaid in the MNI 152 T1 template; the number indicates the MNI coordinates. CA, cornu ammonis; MNI, Montreal Neurologic Institute.

**Figure 2:**
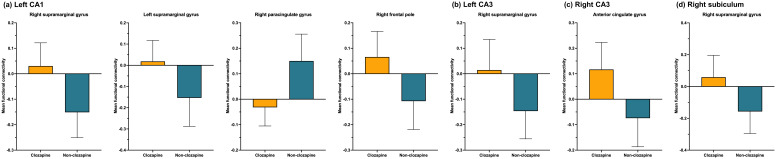
Mean resting-state functional connectivity with the hippocampal subregions. **(A)** Left CA1, **(B)** Left CA3, **(C)** Right CA3, **(D)** Right subiculum. The orange and blue bars indicate clozapine and non-clozapine groups, respectively. The error bars indicate standard deviations. CA, cornu ammonis.

**Figure 1** and **Table 3** present seven clusters exhibiting significant group differences in functional connectivity with the hippocampal subregions. While the cluster located in the right paracingulate gyrus showed decreased functional connectivity with the left CA1 in the clozapine group compared with the non-clozapine group, the other clusters exhibited increased functional connectivity with their corresponding seed regions in the clozapine group. **Figure 2** illustrates the mean functional connectivity of these clusters with their corresponding seed regions in the non-clozapine and clozapine groups.

**Table 3:** Clusters exhibiting group differences in resting-state functional connectivity with the hippocampal subregions.

Seed	Region	MNI coordinate			Cluster size	Cluster FDR p
		x	Y	z		
Left CA1	Right supramarginal gyrus	+54	-36	+38	1,080	<0.001
Left CA1	Left supramarginal gyrus	-64	-22	+42	421	<0.001
Left CA1	Right paracingulate gyrus	+10	+46	+18	324	0.001
Left CA1	Right frontal pole	+46	+42	+18	192	0.010
Left CA3	Right supramarginal gyrus	+48	-46	+60	384	<0.001
Right CA3	Anterior cingulate gyrus	+04	+02	+30	162	0.048
Right subiculum	Right supramarginal gyrus	+60	-42	+44	432	<0.001

CA, cornu ammonis; FDR, false discovery rate; MNI, Montreal Neurologic Institute.

### 3.3 Clinical associations of structural and resting-state functional alterations in hippocampal subregions

**Figure 3:**
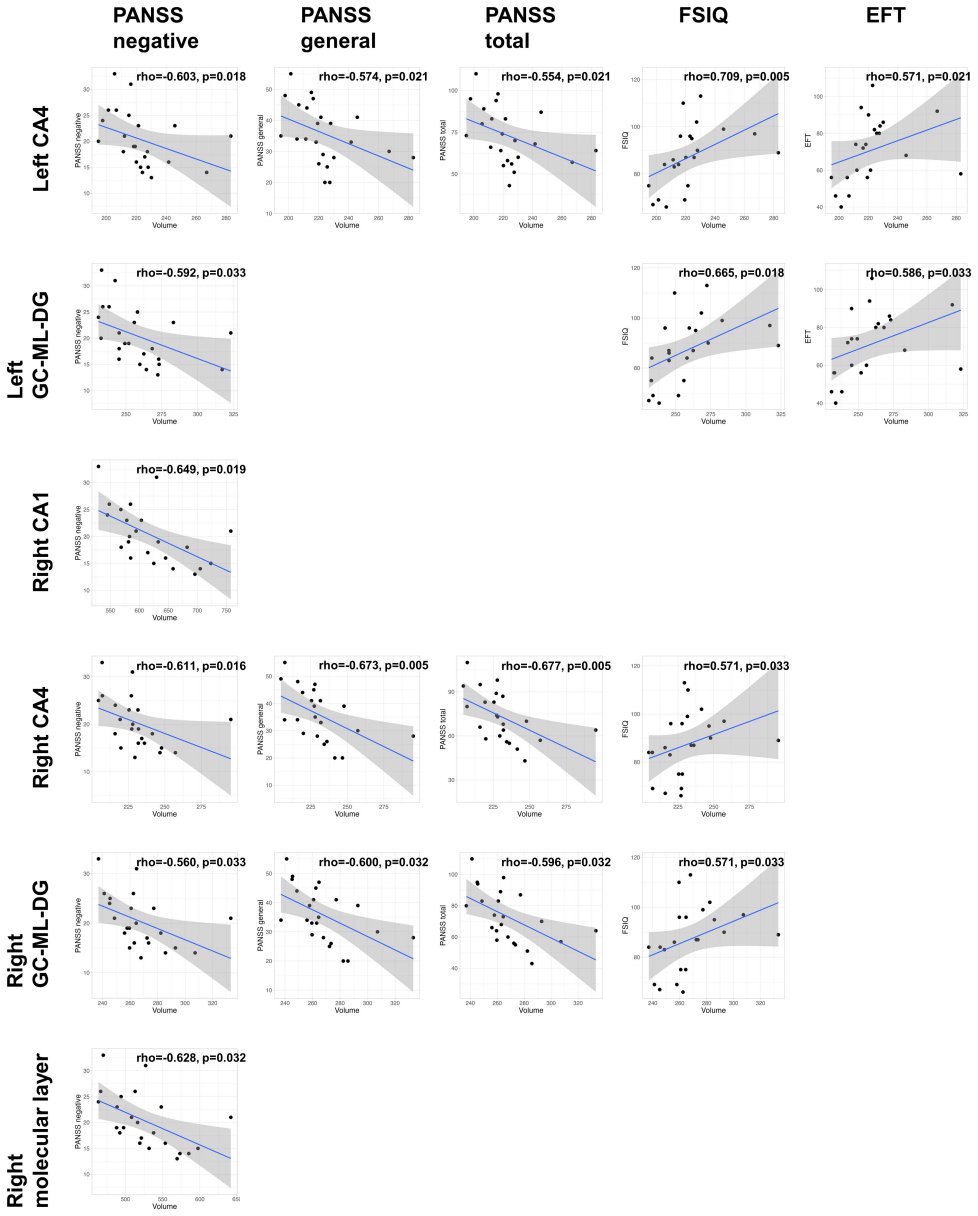
Clinical associations of the volumes of hippocampal subregions in the clozapine group. The p-values were adjusted based on a false discovery rate (FDR) q of 0.05. CA, cornu ammonis; GC-ML-DG, Granule Cell and Molecular Layer of the Dentate Gyrus; PANSS, positive and negative syndrome scale; FSIQ, full-scale intelligence quotient; EFT, executive function test.

In the non-clozapine group, we did not observe any associations between the volumes of hippocampal subregions and clinical variables (**Supplementary Table 3**). **Supplementary Table 4** and **Figure 3** show the associations of volumes of hippocampal subregions with clinical variables in the clozapine group. The volume of the left CA4 was associated with the PANSS negative subscale (rho=-0.603, FDR p=0.018), general subscale (rho=-0.574, FDR p=0.021), total (rho=-0.554, FDR p=0.021), FSIQ (rho=0.709, FDR p=0.005), and EFQ scores (rho=0.571, FDR p=0.021). The volume of the left GC-ML-DG correlated with the PANSS negative subscale (rho=-0.592, FDR p=0.033), FSIQ (rho=0.665, FDR p=0.018), and EFQ (rho=0.586, FDR p=0.033) scores. Associations of the volumes of the right CA4 and GC-ML-DG with the PANSS negative subscale (CA4: rho=-0.611, FDR p=0.016; GC-ML-DG: rho=-0.560, FDR p=0.033), general subscale (CA4: rho=-0.673, FDR p=0.005; GC-ML-DG: rho=-0.600, FDR p=0.032), total (CA4: rho=-0.677, FDR p=0.005; GC-ML-DG: rho=-0.596, FDR p=0.032), and FSIQ (CA4: rho=0.571, FDR p=0.033; GC-ML-DG: rho=0.571, FDR p=0.033) scores were observed. The PANSS negative subscale negatively correlated with the volumes of the right CA1 (rho=-0.649, FDR p=0.019) and molecular layer (rho=-0.628, FDR p=0.032).

**Supplementary Tables 5** and **6** show the clinical associations of functional connectivity with the hippocampal subregions in the non-clozapine and clozapine groups. In the clozapine group, the functional connectivity between the left CA3 and the cluster in the right supramarginal gyrus was positively correlated with the FSIQ (rho=0.737, FDR p=0.003). We observed a positive association in functional connectivity between the right CA3 and the cluster in the anterior cingulate gyrus, with the PANSS negative subscale score (rho=0.605, FDR p=0.049).

No significant association between the structural and functional alterations of the hippocampal subregions and illness duration (**Supplementary Tables 7** and **8**) or clozapine dose (**Supplementary Tables 9** and **10**) was observed.

## 4 Discussion

In the present study, we included patients with schizophrenia whose illness duration was > 5 years and categorized them according to whether they had taken clozapine at the date of the MRI scan. Group comparisons of structural and resting-state functional abnormalities of the hippocampus were performed. Compared with the non-clozapine group, the clozapine group had smaller volumes of the hippocampus and several hippocampal subregions, including CA1, CA4, GC-ML-DG, and the molecular layer in both hemispheres. Seven clusters showed significant group differences in functional connectivity with their respective seed regions, with six of the clusters exhibiting increased functional connectivity in the clozapine group. The smaller volumes of hippocampal subregions in the clozapine group were associated with clinical variables, including the PANSS total, negative subscale, general subscale, FSIQ, and EFQ scores, and the magnitude of clinical associations was moderate. In the clozapine group, the functional connectivity between the left CA3 and the cluster in the right supramarginal gyrus was positively correlated with the FSIQ. Additionally, the functional connectiviy between the right CA3 and anterior cingulate gyrus had a positive association with the PANSS negative subscale score. This cross-sectional study revealed structural and resting-state functional abnormalities of the hippocampus associated with clozapine treatment in schizophrenia. Notably, the smaller volume of hippocampal subregions in the clozapine group had moderate correlations with poorer clinical outcomes.

We observed volume decreases in the hippocampus and hippocampal subregions in the clozapine group compared with the non-clozapine group. Volume loss of the hippocampus in schizophrenia compared to healthy controls was demonstrated in a large-scale previous study, showing the largest effect size among subcortical brain regions (2). In patients with treatment resistance who initiated clozapine treatment, reduction in the volume of the hippocampus has not been consistently reported. Tronchin et al. showed that after 6 months of clozapine treatment, patients with treatment-resistant schizophrenia had smaller volumes of the hippocampus, thalamus, caudate, and putamen compared with healthy controls (20). However, Krajner et al. did not observe reduced hippocampal volume in patients with treatment-resistant schizophrenia after 12 weeks of clozapine treatment (28). According to a review of structural MRI studies on hippocampal subregion abnormalities in schizophrenia (29), volume decrease is prominent in CA1, CA2/3, CA4, dentate gyrus, and subiculum subregions regardless of hemisphere specificity. Despite variations in individual findings across studies, existing evidence supports that hippocampal subregion volumes are progressively reduced from prodromal to chronic stages of the illness and that the CA1 may be a target subregion affected by the illness in the prodromal and early stages (29, 30). Several previous studies have reported the associations of structural changes in hippocampal subregions with antipsychotic treatment in patients with first-episode psychosis (31–33). However, the association with clozapine treatment is still uncertain, mainly due to a very limited number of previous studies on this issue. Our study showed smaller volumes of the hippocampal subregions in patients undergoing clozapine treatment compared with those not taking clozapine. Further studies are necessary to evaluate the impacts of clozapine treatment on widespread structural changes in the hippocampus in chronic schizophrenia.

We observed several group differences in the functional connectivity between the hippocampal subregions, with the majority showing increased functional connectivity in the clozapine group. Regarding alterations in resting-state functional connectivity of the hippocampus in schizophrenia, Zhou et al. showed reduced functional connectivity of bilateral hippocampi to brain regions involved in episodic memory in patients with schizophrenia compared with healthy controls (34). Additionally, the functional dysconnectivity of the hippocampus in schizophrenia has been previously reported (21, 22); however, no prior studies have shown resting-state functional abnormalities of the hippocampus in association with clozapine treatment in schizophrenia. The right supramarginal gyrus, which exhibited increased functional connectivity with the left CA1, CA3, and right subiculum in our findings, is associated with social judgment (35), empathy (36), and understanding others’ internal states (37). Previous studies have reported on the implications of structural and functional impairments of the supramarginal gyrus in the pathophysiology of schizophrenia (38–40). The anterior cingulate cortex is involved in emotion, motivation, and cognition, with connections to both the limbic system and prefrontal cortex (41, 42). Many previous MRI studies have revealed structural and functional alterations of the anterior cingulate along with their clinical associations in schizophrenia (43–47). Paracingulate sulcus morphology is associated with hallucinations (48) and executive function (49) in patients with schizophrenia, and the frontal pole has emerged as a key region in the pathophysiology of schizophrenia (50) in relation to cognitive insight (51). Despite abundant evidence on the abnormalities of each brain region included in the dysconnectivity of the hippocampal subregions observed in our findings, functional dysconnectivity between these brain regions has been scarcely reported. We also identified significant associations between the functional connectivity of the hippocampal subregions and both the FSIQ and the PANSS negative subscale score in the clozapine group. Our results provide exploratory evidence of the implications of altered functional connectivity of the hippocampal subregions for the clinical symptoms of schizophrenia. However, further studies are needed to clarify the role of functional abnormalities in brain regions in the pathophysiology of schizophrenia, particularly in relation to clozapine treatment.

We showed that the reduced volumes of hippocampal subregions in the clozapine group had moderate associations with several clinical variables related to the severity of psychiatric symptoms and cognitive functions. According to a selective review by Hu et al., volumes of various hippocampal subregions are significantly associated with symptom severity, usually measured by the PANSS (29). The clinical associations between the volumes of hippocampal subregions and the severity of positive or negative symptoms, depending on individual studies. In this study, the associations were mainly observed between the volumes of CA4 and GC-ML-DG subregions and the PANSS negative subscale score. This may indicate neurobiological markers reflecting the severity of negative symptoms in patients with chronic schizophrenia. Regarding associations with cognitive functions, we found a relationship between the volumes of the left CA4 and GC-ML-DG subregions and general intelligence and executive function. Similar to previous findings regarding associations with the PANSS score, the findings for correlations with cognitive functions also varied across individual studies (29). Notably, the clinical associations in our findings were moderate, showing a stronger magnitude than reported in previous studies. This might be due to the clinically homogeneous characteristics of the study population, i.e., clozapine treatment, given that no significant associations were noted in the non-clozapine group. We did not clarify how the individual subregions were involved in clinical symptoms, which should be elucidated in future studies. Further, a longitudinal study is needed to disentangle associations of the structural changes in these hippocampal subregions with clinical symptoms in relation to clozapine treatment.

Despite several notable findings of the current study, we acknowledge some limitations. First, the relatively small sample size may have limited the statistical power. It is necessary for our findings to be replicated with a larger sample size. Second, in the non-clozapine group, we identified one patient with a history of clozapine treatment. Although the reason for initiating and terminating the clozapine treatment was uncertain, the patient had been stabilized without relapses after discontinuing the medication for a few years. However, the patient might be labeled as treatment-resistant, suggesting that it may be inappropriate to interpret our findings in terms of treatment resistance. Furthermore, due to recall bias and the long duration of illnesses among participants, we did not categorize them based on treatment resistance to first-line antipsychotics. This issue prevents one from drawing any conclusions regarding treatment resistance as a factor in our results. Third, due to the cross-sectional study design, whether clozapine treatment induced the decreased volumes of the hippocampus in patients with schizophrenia remains uncertain. In other words, the structural and functional abnormalities of the hippocampus in the clozapine group may have been related to clozapine treatment, or they may have been due to underlying neurobiological changes. Inconsistent findings have been reported regarding this issue (20, 28), which should be clarified by a long-term longitudinal study with sufficient participants. Fourth, we compared structural and functional abnormalities of the hippocampus between patients receiving clozapine and those who were not. The absence of healthy controls in this study limits the interpretation of our findings, particularly when compared to previous studies that included healthy controls. Future research should incorporate healthy controls to validate and further explore on our findings.

## 5 Conclusions

Here, we investigated structural and resting-state functional abnormalities of the hippocampus in chronic schizophrenia categorized by clozapine treatment. The clozapine group showed decreased volumes of the hippocampus and hippocampal subregions, including CA1, CA4, GC-ML-DG, and the molecular layer compared to the non-clozapine group. In the clozapine group, the reduced volumes of CA4 and GC-ML-DG were moderately associated with the severity of negative symptoms, general intelligence, and executive function. Seven clusters were identified as having significant group differences in functional connectivity with the hippocampal subregions, with six of the clusters showing increased functional connectivity in the clozapine group. We recruited patients with schizophrenia whose illness duration was > 5 years and reported structural changes of the hippocampus moderately associated with clinical variables in the patients undergoing clozapine treatment. A clinically homogeneous population would be beneficial to reveal disease-specific alterations of the brain, despite promising results from early psychosis studies. Our findings, especially the associations of reduced volumes of the hippocampal subregions with clinical symptoms in the clozapine group, should be validated in future studies with a longitudinal study design.

## Data Availability

The raw data supporting the conclusions of this article will be made available by the authors, without undue reservation.
