# Supplementary material for: Association of clozapine with structural and resting-state functional abnormalities of the hippocampus in chronic schizophrenia
Source: Front Psychiatry. 2024 Oct 4;15:1464066. doi: 10.3389/fpsyt.2024.1464066 (PMC11486750; doi:10.3389/fpsyt.2024.1464066)
Supplement: Supplementary file 1 [file DataSheet1.docx]

**Supplementary Material**

**Supplementary Table 1.** Information on image parameters

|  | T1-weighted image | Resting-state functional MR image^b^ |
| --- | --- | --- |
| Sequences | 3D, FFE | EPI |
| TE (ms) | 2.9 | 30 |
| TR (ms) | 6.5 | 3000 |
| Flip angle (º) | 9 | 90 |
| FOV (mm) (RL, AP, FH)^a^ | 211, 256, 256 | 212, 199, 159 |
| Voxel size (mm) | 1.0, 1.0, 1.0 | 3.3, 3.3, 3.3 |
| Number of slices | 211 | 48 |
| Slice orientation | Sagittal | Axial |
| Slice thickness (mm) | 1 | 3.3 |

^a^ Right/left; anterior/posterior; and foot/head.

^b^ Interleaved order (1, 4, 7 … 6, 9).

MR: magnetic resonance; FFE: fast field echo; EPI: echo-planar imaging; TE: echo time; TR: repetition time; FOV: field of view.

**Supplementary Table 2.** Group differences in volumes of subcortical regions

|  | Non-clozapine | Clozapine |  |  |  |
| --- | --- | --- | --- | --- | --- |
| Region | Volume^a^ | | t | Uncorrected p | FDR p |
| Left accumbens | 549.1 (144.1) | 501.0 (82.2) | -1.665 | 0.103 | 0.190 |
| Left amygdala | 1637.9 (208.3) | 1516.1 (189.6) | -2.449 | 0.018 | 0.085 |
| Left caudate | 3360.0 (395.2) | 3238.3 (344.6) | -1.066 | 0.292 | 0.372 |
| Left hippocampus | 3966.1 (454.1) | 3618.5 (340.8) | -3.009 | 0.004 | 0.029 |
| Left pallidum | 2060.4 (244.7) | 2018.1 (255.5) | -0.613 | 0.543 | 0.543 |
| Left putamen | 4998.2 (632.6) | 4854.0 (466.5) | -1.416 | 0.163 | 0.229 |
| Left thalamus | 7465.5 (988.9) | 7305.6 (806.7) | -0.731 | 0.469 | 0.506 |
| Right accumbens | 590.2 (114.0) | 528.1 (69.4) | -2.256 | 0.029 | 0.101 |
| Right amygdala | 1774.7 (246.1) | 1656.3 (194.4) | -1.622 | 0.112 | 0.190 |
| Right caudate | 3562.8 (288.3) | 3385.4 (348.7) | -2.109 | 0.040 | 0.113 |
| Right hippocampus | 4030.8 (454.9) | 3703.8 (345.7) | -3.063 | 0.004 | 0.029 |
| Right pallidum | 2114.2 (235.7) | 2067.8 (243.8) | -0.729 | 0.470 | 0.506 |
| Right putamen | 5095.8 (502.5) | 4931.7 (460.3) | -1.921 | 0.061 | 0.142 |
| Right thalamus | 6904.4 (753.4) | 6670.0 (742.6) | -1.576 | 0.122 | 0.190 |

^a^ Volumes of subcortical regions are presented as mean (SD).

FDR: false discovery rate.

**Supplementary Table 3.** Clinical associations of volumes of hippocampal subregions in the non-clozapine group

|  | Left CA1 | | Left CA4 | | Left GC-ML-DG | | Left molecular layer | | Right CA1 | | Right CA4 | | Right GC-ML-DG | | Right molecular layer | |
| --- | --- | --- | --- | --- | --- | --- | --- | --- | --- | --- | --- | --- | --- | --- | --- | --- |
| Clinical variable | rho | FDR p | rho | FDR p | rho | FDR p | rho | FDR p | rho | FDR p | rho | FDR p | rho | FDR p | rho | FDR p |
| PANSS positive | -0.017 | 0.969 | -0.072 | 0.898 | -0.029 | 0.993 | -0.073 | 0.787 | -0.124 | 0.660 | -0.014 | 0.944 | -0.043 | 0.898 | -0.097 | 0.706 |
| PANSS negative | 0.108 | 0.737 | -0.010 | 0.959 | 0.020 | 0.993 | 0.087 | 0.787 | 0.083 | 0.698 | 0.070 | 0.860 | 0.088 | 0.819 | 0.063 | 0.812 |
| PANSS general | 0.105 | 0.737 | -0.012 | 0.959 | -0.002 | 0.993 | 0.078 | 0.787 | 0.189 | 0.520 | 0.044 | 0.896 | 0.044 | 0.898 | 0.099 | 0.706 |
| PANSS total | 0.066 | 0.799 | -0.029 | 0.959 | -0.007 | 0.993 | 0.039 | 0.877 | 0.093 | 0.688 | 0.045 | 0.896 | 0.044 | 0.898 | 0.039 | 0.877 |
| GAF | -0.096 | 0.737 | 0.018 | 0.959 | -0.031 | 0.993 | -0.069 | 0.787 | -0.095 | 0.688 | -0.118 | 0.723 | -0.099 | 0.813 | -0.099 | 0.706 |
| FSIQ | 0.238 | 0.320 | 0.292 | 0.213 | 0.297 | 0.257 | 0.252 | 0.371 | 0.149 | 0.622 | 0.249 | 0.308 | 0.235 | 0.330 | 0.193 | 0.506 |
| MQ | 0.008 | 0.969 | 0.018 | 0.959 | -0.024 | 0.993 | -0.007 | 0.973 | -0.014 | 0.942 | 0.084 | 0.842 | 0.032 | 0.906 | 0.010 | 0.959 |
| EFQ | 0.272 | 0.264 | 0.302 | 0.205 | 0.263 | 0.311 | 0.245 | 0.371 | 0.172 | 0.558 | 0.305 | 0.204 | 0.250 | 0.305 | 0.183 | 0.511 |

CA: cornu ammonis, GC-ML-DG: granule cell and molecular layer of the dentate gyrus, FDR: false-discovery rate, PANSS: positive and negative syndrome scale, GAF: global assessment of functioning, FSIQ: full-scale intelligence quotient, MQ: memory quotient, EFQ: executive function quotient.

**Supplementary Table 4.** Clinical associations of volumes of hippocampal subregions in the clozapine group

|  | Left CA1 | | Left CA4 | | Left GC-ML-DG | | Left molecular layer | | Right CA1 | | Right CA4 | | Right GC-ML-DG | | Right molecular layer | |
| --- | --- | --- | --- | --- | --- | --- | --- | --- | --- | --- | --- | --- | --- | --- | --- | --- |
| Clinical variable | rho | FDR p | rho | FDR p | rho | FDR p | rho | FDR p | rho | FDR p | rho | FDR p | rho | FDR p | rho | FDR p |
| PANSS positive | -0.443 | 0.099 | -0.216 | 0.484 | -0.185 | 0.595 | -0.208 | 0.489 | -0.423 | 0.133 | -0.471 | 0.080 | -0.325 | 0.260 | -0.322 | 0.279 |
| PANSS negative | -0.522 | 0.071 | -0.603 | 0.018 | -0.592 | 0.033 | -0.503 | 0.173 | -0.649 | 0.019 | -0.611 | 0.016 | -0.560 | 0.033 | -0.628 | 0.032 |
| PANSS general | -0.458 | 0.096 | -0.574 | 0.021 | -0.493 | 0.057 | -0.365 | 0.287 | -0.516 | 0.071 | -0.673 | 0.005 | -0.600 | 0.032 | -0.495 | 0.079 |
| PANSS total | -0.509 | 0.071 | -0.554 | 0.021 | -0.503 | 0.057 | -0.404 | 0.224 | -0.565 | 0.059 | -0.677 | 0.005 | -0.596 | 0.032 | -0.534 | 0.079 |
| GAF | 0.374 | 0.190 | 0.246 | 0.413 | 0.227 | 0.474 | 0.286 | 0.371 | 0.398 | 0.144 | 0.431 | 0.120 | 0.431 | 0.135 | 0.440 | 0.143 |
| FSIQ | 0.585 | 0.067 | 0.709 | 0.005 | 0.665 | 0.018 | 0.453 | 0.206 | 0.493 | 0.076 | 0.571 | 0.033 | 0.571 | 0.033 | 0.317 | 0.279 |
| MQ | 0.107 | 0.737 | 0.146 | 0.702 | 0.115 | 0.856 | 0.208 | 0.489 | 0.130 | 0.688 | -0.182 | 0.608 | -0.154 | 0.712 | 0.185 | 0.561 |
| EFQ | 0.447 | 0.099 | 0.571 | 0.021 | 0.586 | 0.033 | 0.471 | 0.206 | 0.497 | 0.076 | 0.350 | 0.204 | 0.427 | 0.135 | 0.506 | 0.079 |

CA: cornu ammonis, GC-ML-DG: granule cell and molecular layer of the dentate gyrus, FDR: false-discovery rate, PANSS: positive and negative syndrome scale, GAF: global assessment of functioning, FSIQ: full-scale intelligence quotient, MQ: memory quotient, EFQ: executive function quotient.

**Supplementary Table 5.** Clinical associations of resting-state functional connectivity with hippocampal subregions in the non-clozapine group

|  |  | PANSS positive | | PANSS negative | | PANSS general | | PANSS total | | GAF | | FSIQ | | MQ | | EFQ | |
| --- | --- | --- | --- | --- | --- | --- | --- | --- | --- | --- | --- | --- | --- | --- | --- | --- | --- |
| Seed | Region | rho | FDR p | rho | FDR p | rho | FDR p | Rho | FDR p | rho | FDR p | rho | FDR p | rho | FDR p | rho | FDR p |
| Left CA1 | Right supramarginal gyrus | -0.332 | 0.168 | -0.414 | 0.168 | -0.174 | 0.462 | -0.342 | 0.168 | 0.289 | 0.215 | 0.124 | 0.554 | 0.352 | 0.168 | 0.369 | 0.168 |
| Left CA1 | Left supramarginal gyrus | -0.27 | 0.22 | -0.309 | 0.193 | -0.338 | 0.193 | -0.335 | 0.193 | 0.334 | 0.193 | 0.183 | 0.381 | 0.349 | 0.193 | 0.3 | 0.193 |
| Left CA1 | Right paracingulate gyrus | 0.091 | 0.956 | 0.012 | 0.956 | 0.069 | 0.956 | 0.013 | 0.956 | -0.142 | 0.956 | -0.133 | 0.956 | -0.249 | 0.919 | -0.255 | 0.919 |
| Left CA1 | Right frontal pole | -0.019 | 0.927 | 0.029 | 0.927 | 0.213 | 0.817 | 0.066 | 0.927 | -0.041 | 0.927 | -0.023 | 0.927 | 0.322 | 0.817 | 0.256 | 0.817 |
| Left CA3 | Right supramarginal gyrus | -0.388 | 0.164 | -0.187 | 0.423 | -0.335 | 0.202 | -0.379 | 0.164 | 0.124 | 0.553 | 0.228 | 0.365 | 0.458 | 0.164 | 0.282 | 0.274 |
| Right CA3 | Anterior cingulate gyrus | 0.224 | 0.621 | 0.205 | 0.621 | 0.096 | 0.649 | 0.224 | 0.621 | -0.173 | 0.621 | 0.121 | 0.647 | 0.289 | 0.621 | 0.153 | 0.621 |
| Right subiculum | Right supramarginal gyrus | -0.191 | 0.577 | -0.232 | 0.577 | -0.059 | 0.783 | -0.164 | 0.577 | 0.174 | 0.577 | 0.058 | 0.783 | 0.242 | 0.577 | 0.265 | 0.577 |

CA: cornu ammonis, FDR: false-discovery rate, PANSS: positive and negative syndrome scale, GAF: global assessment of functioning, FSIQ: full-scale intelligence quotient, MQ: memory quotient, EFQ: executive function quotient.

**Supplementary Table 6.** Clinical associations of resting-state functional connectivity with hippocampal subregions in the clozapine group

|  |  | PANSS positive | | PANSS negative | | PANSS general | | PANSS total | | GAF | | FSIQ | | MQ | | EFQ | |
| --- | --- | --- | --- | --- | --- | --- | --- | --- | --- | --- | --- | --- | --- | --- | --- | --- | --- |
| Seed | Region | rho | FDR p | rho | FDR p | rho | FDR p | Rho | FDR p | rho | FDR p | rho | FDR p | rho | FDR p | rho | FDR p |
| Left CA1 | Right supramarginal gyrus | 0.087 | 0.722 | -0.272 | 0.671 | -0.204 | 0.671 | -0.197 | 0.671 | 0.278 | 0.671 | 0.142 | 0.720 | 0.122 | 0.72 | 0.31 | 0.671 |
| Left CA1 | Left supramarginal gyrus | -0.077 | 0.862 | -0.18 | 0.645 | -0.214 | 0.645 | -0.171 | 0.645 | 0.343 | 0.645 | 0.174 | 0.645 | -0.328 | 0.645 | -0.019 | 0.937 |
| Left CA1 | Right paracingulate gyrus | 0.212 | 0.767 | -0.119 | 0.838 | -0.016 | 0.974 | 0.008 | 0.974 | -0.246 | 0.767 | 0.334 | 0.703 | 0.334 | 0.703 | 0.16 | 0.82 |
| Left CA1 | Right frontal pole | 0.337 | 0.852 | 0.022 | 0.935 | 0.21 | 0.852 | 0.194 | 0.852 | 0.123 | 0.935 | -0.24 | 0.852 | -0.021 | 0.935 | 0.046 | 0.935 |
| Left CA3 | Right supramarginal gyrus | -0.3 | 0.282 | -0.38 | 0.197 | -0.45 | 0.197 | -0.399 | 0.197 | 0.205 | 0.399 | 0.737 | 0.003 | -0.216 | 0.399 | 0.366 | 0.197 |
| Right CA3 | Anterior cingulate gyrus | 0.309 | 0.316 | 0.605 | 0.049 | 0.417 | 0.152 | 0.482 | 0.098 | -0.263 | 0.368 | -0.492 | 0.098 | -0.026 | 0.919 | -0.22 | 0.418 |
| Right subiculum | Right supramarginal gyrus | -0.174 | 0.622 | -0.173 | 0.622 | -0.149 | 0.622 | -0.167 | 0.622 | -0.061 | 0.805 | 0.543 | 0.13 | 0.198 | 0.622 | 0.391 | 0.393 |

CA: cornu ammonis, FDR: false-discovery rate, PANSS: positive and negative syndrome scale, GAF: global assessment of functioning, FSIQ: full-scale intelligence quotient, MQ: memory quotient, EFQ: executive function quotient.

**Supplementary Table 7.** Clinical associations between hippocampal subregion volumes and illness duration

|  | Non-Clozapine | | | Clozapine | | |
| --- | --- | --- | --- | --- | --- | --- |
| Region | rho | Uncorrected p | FDR p | rho | Uncorrected p | FDR p |
| Left CA1 | 0.04 | 0.836 | 0.955 | -0.077 | 0.728 | 0.993 |
| Left CA4 | -0.105 | 0.589 | 0.868 | 0.165 | 0.451 | 0.993 |
| Left GC-ML-DG | -0.088 | 0.651 | 0.868 | 0.151 | 0.492 | 0.993 |
| Left molecular layer | 0.011 | 0.955 | 0.955 | -0.002 | 0.993 | 0.993 |
| Right CA1 | -0.162 | 0.4 | 0.868 | -0.023 | 0.918 | 0.993 |
| Right CA4 | -0.169 | 0.381 | 0.868 | 0.032 | 0.884 | 0.993 |
| Right GC-ML-DG | -0.16 | 0.406 | 0.868 | 0.210 | 0.336 | 0.993 |
| Right molecular layer | -0.114 | 0.556 | 0.868 | 0.057 | 0.797 | 0.993 |

CA: cornu ammonis, GC-ML-DG: granule cell and molecular layer of the dentate gyrus, FDR: false-discovery rate.

**Supplementary Table 8.** Clinical associations of resting-state functional connectivity with hippocampal subregions and illness duration

|  |  | Non-Clozapine | | | Clozapine | | |
| --- | --- | --- | --- | --- | --- | --- | --- |
| Seed | Region | rho | Uncorrected p | FDR p | rho | Uncorrected p | FDR p |
| Left CA1 | Right supramarginal gyrus | -0.003 | 0.988 | 0.988 | 0.191 | 0.432 | 0.757 |
| Left CA1 | Left supramarginal gyrus | -0.042 | 0.84 | 0.981 | 0.340 | 0.155 | 0.510 |
| Left CA1 | Right paracingulate gyrus | 0.446 | 0.026 | 0.089 | -0.068 | 0.783 | 0.889 |
| Left CA1 | Right frontal pole | 0.177 | 0.398 | 0.697 | 0.102 | 0.678 | 0.889 |
| Left CA3 | Right supramarginal gyrus | 0.331 | 0.106 | 0.248 | 0.296 | 0.219 | 0.510 |
| Right CA3 | Anterior cingulate gyrus | -0.447 | 0.025 | 0.089 | 0.356 | 0.135 | 0.510 |
| Right subiculum | Right supramarginal gyrus | -0.073 | 0.728 | 0.981 | -0.034 | 0.889 | 0.889 |

CA: cornu ammonis, FDR: false-discovery rate.

**Supplementary Table 9.** Clinical associations between hippocampal subregion volumes and clozapine dose

| Region | Rho | Uncorrected p | FDR p |
| --- | --- | --- | --- |
| Left CA1 | -0.299 | 0.165 | 0.331 |
| Left CA4 | -0.123 | 0.577 | 0.803 |
| Left GC-ML-DG | -0.075 | 0.733 | 0.837 |
| Left molecular layer | -0.392 | 0.064 | 0.256 |
| Right CA1 | -0.321 | 0.135 | 0.331 |
| Right CA4 | -0.029 | 0.894 | 0.894 |
| Right GC-ML-DG | -0.115 | 0.602 | 0.803 |
| Right molecular layer | -0.478 | 0.021 | 0.168 |

CA: cornu ammonis, GC-ML-DG: granule cell and molecular layer of the dentate gyrus, FDR: false-discovery rate.

**Supplementary Table 10.** Clinical associations of resting-state functional connectivity with hippocampal subregions with clozapine dose

| Seed | Region | rho | Uncorrected p | FDR p |
| --- | --- | --- | --- | --- |
| Left CA1 | Right supramarginal gyrus | -0.065 | 0.792 | 0.988 |
| Left CA1 | Left supramarginal gyrus | 0.555 | 0.014 | 0.096 |
| Left CA1 | Right paracingulate gyrus | 0.004 | 0.988 | 0.988 |
| Left CA1 | Right frontal pole | -0.131 | 0.592 | 0.988 |
| Left CA3 | Right supramarginal gyrus | 0.32 | 0.182 | 0.425 |
| Right CA3 | Anterior cingulate gyrus | -0.334 | 0.163 | 0.425 |
| Right subiculum | Right supramarginal gyrus | -0.021 | 0.931 | 0.988 |

CA: cornu ammonis, FDR: false-discovery rate.


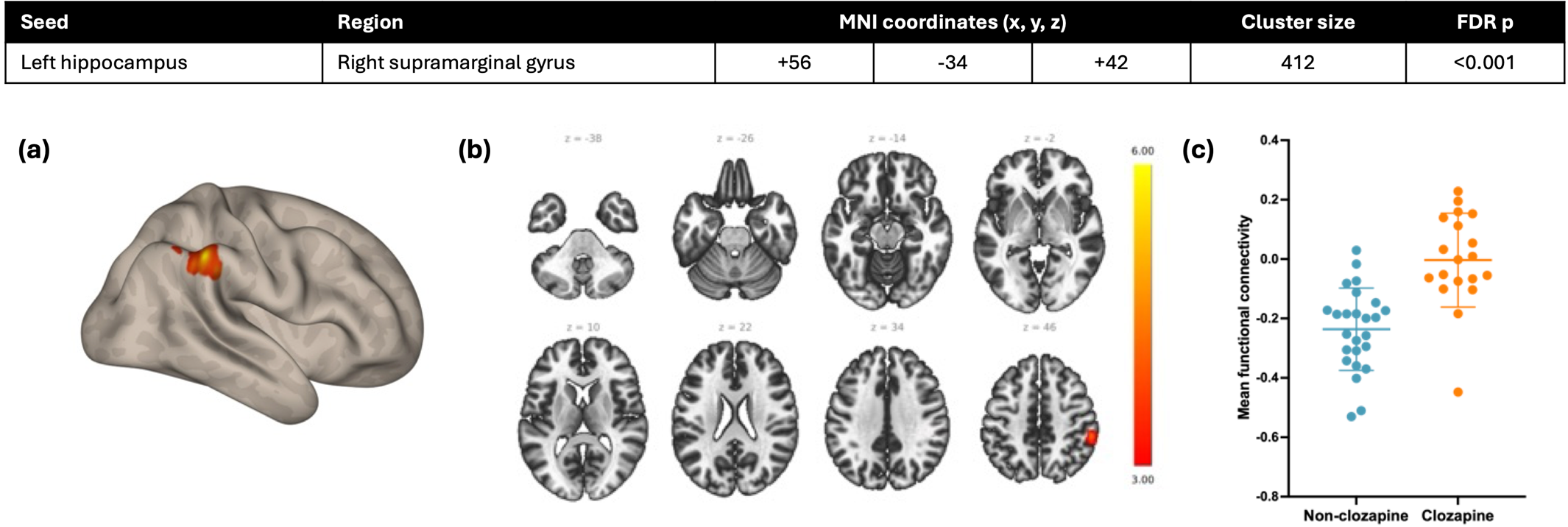


**Supplementary Figure 1. Group differences in resting-state functional connectivity with the left hippocampus between the non-clozapine and clozapine groups.** (a) Right view, (b) Axial view, (c) The blue and orange dots represent the mean functional connectivity values of the cluster in the non-clozapine and clozapine groups, respectively. The blue and orange bars indicate the mean ± standard deviation in the non-clozapine and clozapine groups, respectively.


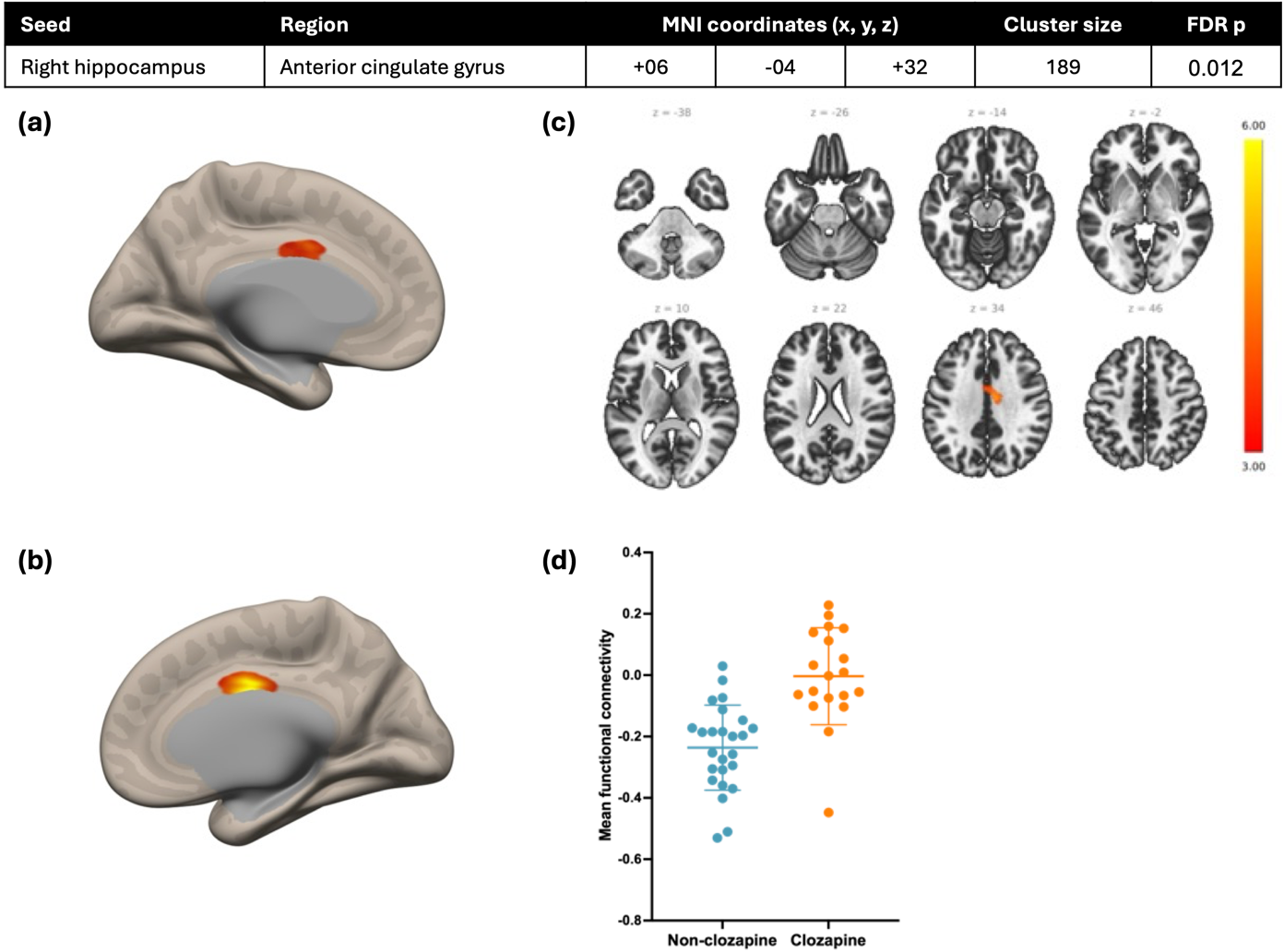


**Supplementary Figure 2. Group differences in resting-state functional connectivity with the right hippocampus.** (a) Left medial view, (b) Right medial view, (c) Axial view, (d) The blue and orange dots represent the mean functional connectivity values of the cluster in the non-clozapine and clozapine groups, respectively. The blue and orange bars indicate the mean ± standard deviation in the non-clozapine and clozapine groups, respectively.
